# EXPERIENCES WITH SHARED DECISION-MAKING AND ADVANCED CARE PLANNING AMONG OLDER SPANISH-SPEAKING LATINOS

**DOI:** 10.1093/geroni/igz038.3517

**Published:** 2019-11-08

**Authors:** Yelba M Castellon-Lopez, Gery Ryan, O Kenrik Duru, Gerardo Moreno

**Affiliations:** 1 University of California, Los Angeles, Los Angeles, California, United States; 2 RAND, Santa Monica, California, United States

## Abstract

Advance care planning (ACP) helps individuals clarify their values and preferences for future care, and to communicate their wishes to loved ones, surrogate decision-makers and healthcare providers in advance of when they become unable to make healthcare decisions. Older Spanish-speaking Latino adults have among the lowest rates of advance directives a form of ACP. Shared decision-making (SDM) interventions have significantly improved outcomes for disadvantaged patients. SDM has proven to be particularly beneficial in ethnic minority populations with low literacy and low socioeconomic status groups. The aim of this study was to pilot an online SDM module delivered in Spanish to better understand experiences with ACP among older Spanish-speaking Latino adults. We recruited a sample of older Latino adults ages 56-81 who were low-income and primarily Spanish-speaking (N=20). Sixty-five percent of the sample was female. Participants were asked to complete an SDM module delivered by a physician to discuss ACP. The online module was developed by Healthwise © a national leader in SDM. Qualitative interviews were conducted to examine experiences with SDM and ACP. SDM improved awareness about ACP options and facilitated conversations with family members among participants. Results from the study demonstrated a four-step process for engaging participants in ACP including: Awareness, Initial conversations, Conversations with medical provider, and Formalizing. Interviews informed opportunities for culturally tailored interventions along different points on the continuum. This study highlights important opportunities to better understand the process-based steps and targeted interventions needed to address disparities in ACP among Spanish-speaking low-income Latinos.

